# Prediction of normalized signal strength on DNA sequencing microarrays by n-grams within a neural network model

**DOI:** 10.6026/97320630015388

**Published:** 2019-05-30

**Authors:** Charles Chilaka, Steven Carr, Nabil Shalaby, Wolfgang Banzhaf

**Affiliations:** 1 1Program in Scientific Computing, Memorial University of Newfoundland and St. John's, Newfoundland, Canada A1C 5S7; 2 2Department of Biology, Memorial University of Newfoundland and St. John's, Newfoundland, Canada A1C 5S7; 3 3Department of Computer Science, Memorial University of Newfoundland and St. John's, Newfoundland, Canada A1C 5S7; 4 4Department of Mathematics and Statistics,Memorial University of Newfoundland and St. John's, Newfoundland, Canada A1C 5S7; 5 5Department of Mathematics, FUT, Owerri,Nigeria; 6 Department of Computer Science and Engineering, Michigan State University, East Lansing MI 48824

**Keywords:** Neural networks, n-grams, Performance, Regression values, Confusion matrix, Receiver Operating Characteristic curves

## Abstract

We have shown previously that a feed-forward, back propagation neural network model based on composite n-grams can predict normalized signal strengths of a microarray based DNA sequencing experiment. The microarray comprises a 4xN set of 25-base single-stranded DNA molecule ('oligos'), specific for each of the four possible bases (A, C, G, or T) for Adenine, Cytosine, Guanine and Thymine respectively at each of N positions in the experimental DNA. Strength of binding between reference oligos and experimental DNA varies according to base complementarity and the strongest signal in any quartet should `call the base` at that position. Variation in base composition of and (or) order within oligos can affect accuracy and (or) confidence of base calls. To evaluate the effect of order, we present oligos as n-gram neural input vectors of degree 3 and measure their performance. Microarray signal intensity data were divided into training, validation and testing sets. Regression values obtained were >99.80% overall with very low mean square errors that transform to high best validation performance values. Pattern recognition results showed high percentage confusion matrix values along the diagonal and receiver operating characteristic curves were clustered in the upper left corner, both indices of good predictive performance. Higher order n-grams are expected to produce even better predictions.

## Background

**Figure 1:**
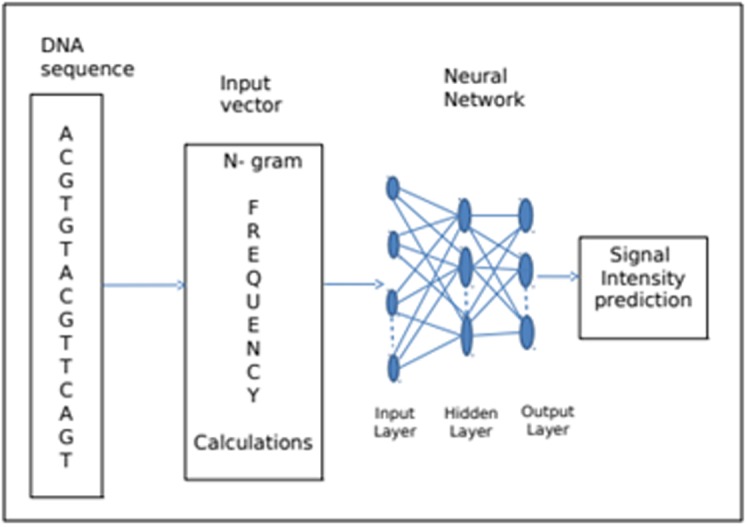
A neural network system for signal intensity prediction. The DNA sequence data are first converted into n-gram profiles as input vectors. The neural network then predicts the normalized signal intensities after network training

DNA sequences are strings of hundreds to millions of four nitrogenous bases (Adenine, Cytosine, Guanine and Thymine) represented by the letters A,C,G, and T respectively. Representation of these strings as numerical values enables the application of powerful dig- ital signal processing techniques. Desirable properties of a DNA numerical representations and some examples are given in 3, 15. N-gram method was first introduced by C.E Shannon in 1948 9. Neural network learning methods provide a robust approach to approximation of realvalued, discrete-valued and vector-valued target functions, 12 such as numerical DNA data. The study of artificial neural networks has been inspired by the observation that biological learning systems are built of very complex webs of interconnected neurons, 10, 11, 12, which communicate through a large set of interconnections assigned variable strengths (weights) in which the learned information is stored, 13. Each neuron computes a weighted sum of its y input signals. The activation function for neurons is the sigmoid function defined in 12 as follows:

σ(y) = 1/1-e^-y^

where y is the weighted sum of the inputs. The output of the sigmoid function ranges from 0 to 1, increasing monotonically with its input and the weights of the interconnections between the different neurons are adjusted during the training process to achieve a desired input/output mapping. Artificial neural networks studies have been used for computational analysis of human DNA sequence 14, single base pair discrimination of terminal mismatches 16, biological phenomena through computational intelligence 17, human donor and acceptor sites prediction 18, coding region recognition and gene identification 19, predicting transmembrane domains of proteins 20 and the prediction of nucleotide sequences using genomic signals 21. We previously 15 used mainly 1-gram, 2-gram and their composition to predict normalized signal strengths from a DNA-sequencing microarray. Here, numerical data from an Affymetrix 1 DNA re-sequencing experiment are normalized and partitioned into training, testing and validation set within a Matlab 2 neural network with 4, 16, 64 for mono-, di-, and tri-nucleotide strings respectively in the input layer. The influence of the length of oligo nucleotide in the nucleotide hybridization intensity experiments is examined by replacement of mono-, di- and tri nucleotide strings with their respective n-gram equivalents. The n-gram ratios for mono-, di- and tri nucleotides are shown in Table 1, Table 2 and Table 3 respectively. The results with 1-gram and 2-gram and their composition have been discussed in 15. We advance the results obtained previously by examining the influence of 3- grams on overall performance of our predictions based on the data evaluation functions. We examine the effect of the different number of neurons in the hidden layer on optimal prediction performance. The output node layer has 4 nodes reflecting our choice of sequence signals to predict. The schematics of DNA neural network architecture are shown in Figure 1. The DNA sequence data are first converted by a sequence encoding schema into neural network input vectors (ratios of n-gram). The neural network then predicts those normalized intensities according to the sequence information embedded in the neural interconnections after network training. 

### Data evaluation functions:

In 15, we explained the concept of performance and regression values. We also examined their results using 1-gram, 2-gram and their composition. We now check for consistency of the results with the inclusion of 3-gram using two other Matlab neural network data evaluation functions. Performance and regression values are also considered with this inclusion.

### Confusion Matrix:

**Figure 2:**
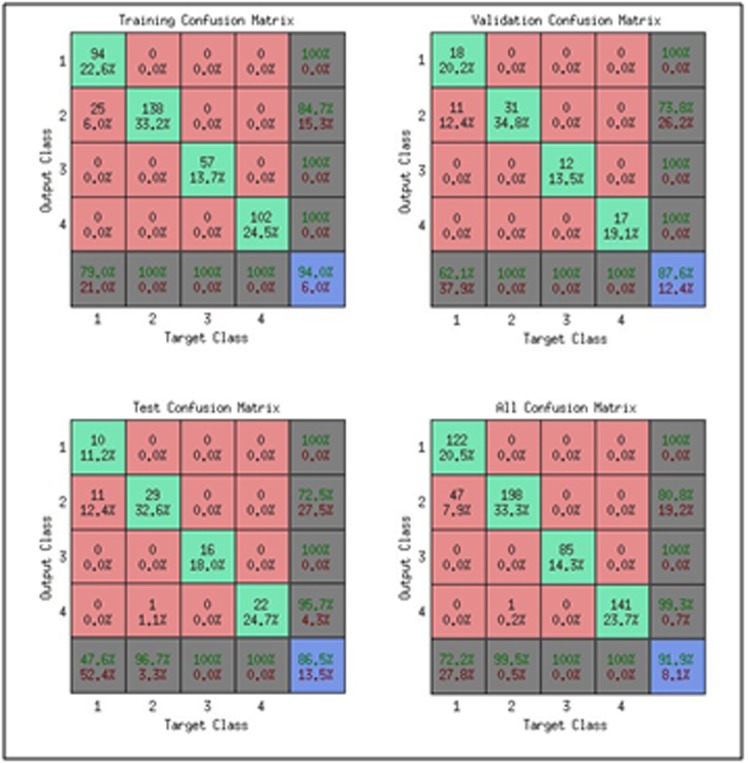
A typical confusion matrix showing various types of errors that occurred for the final trained network.

This is a 2-dimensional matrix with a row and column for each class for training, validation, testing and all datasets. Each matrix element shows the number of test examples for which the actual class is the row and the predicted class is the column. Good results correspond to large numbers down the main diagonal. The diagonal (green cells) in each table show the number of cases that were correctly classified. The off-diagonal (red cells) show the misclassified cases. Blue cells in the bottom right show the total percent of correctly classified cases (in green text) and the total percent of misclassified cases (in red text). Figure 2 shows a confusion matrix with 4 tables each displaying the network response for the training, validation, testing and all datasets.

### Receiver Operating Characteristic, ROC:

**Figure 3:**
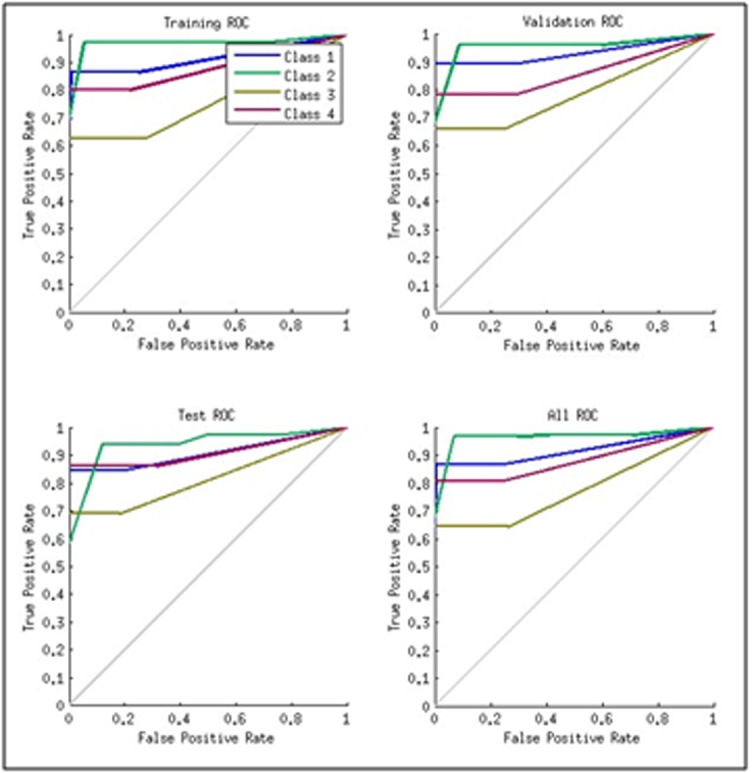
A typical ROC plot

ROC curve shown in Figure 3 is another form of visualization and analysis of the quality of our network. It is a plot of true positive rate (sensitivity) versus the false positive rate (1- specificity). The colored lines in each axis represent the ROC curves for each category of the problem. The ROC always goes through the origin and through (1, 1). A good test would show points in the upper-left corner.

## Methodology

We adopt the same methods as in 15. The dataset is from the Cambridge Reference Sequence with ascension number NC-012920 and is made of 15,453 rows and 6 columns where 3 of the columns are the n-grams for n= 1,2, 3 and the other 4 columns represent the normalized intensities for Adenine, Cytosine, Guanine and Thymine. We extract every 26th line of the dataset which reduces the dataset to 594 rows (lines) respectively. 3-gram are used independently to predict the normalized intensities for the four nucleotides ACGT and results obtained are compared with those obtained in 15. We also use 1-3- gram, 2-3- gram and 1-2-3-gram to repeat the analysis and compare with earlier results.

### The algorithmic steps for our data manipulation are as follows:

1.Compute n-gram profiles of the DNA data set using Python programming language.

2.Calculate the nucleotide, di nucleotide and tri nucleotide frequencies of these profiles..

3.Do substitution of the nucleotides, di nucleotides and tri nucleotide strings with their respective frequencies. Do the following on the intensity profiles:

4. Calculate the highest and lowest value along each row..

5. Do normalization along each row using N(i) = (yi - min)/(max - min), where yi is the actual value of the attribute i, max and min are the maximum and minimum values along each row..

6. Repeat step 5 for every row of intensity profile..

7. Combine results obtained from step 1 to step 6..

8. Extract every 26th line (to avoid subsequence overlap and possibility of random match) from the data set after the operations above..

9. Use Matlab subroutines to get performance plots, regression values, confusion matrices and Receiver operating characteristic curves.

**Figure 4:**
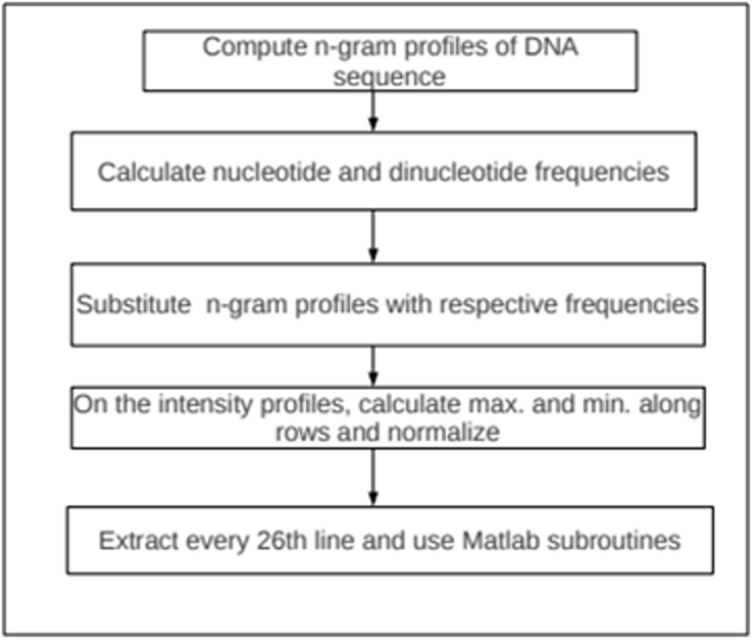
Algorithmic flowchart for computing n-gram profiles and doing normalization on the DNA sequence

The flowchart for the steps is shown in Figure 4.

## Results

Neural network regression value R, determine how robust the prediction is. Higher R value and a smaller MSE in terms of performance imply good prediction. We compare the performances of the networks with 1-3-gram, 2-3-gram and 1-2-3- gram with different number of neurons in the hidden layer using the Matlab regression toolkit. These results are compared with those obtained in 15. Again, the number of neurons in the hidden layer has been varied between 20 and 40 with step size 5 as a matter of choice and hopefully to find the optimal network architecture. Table 7 gives a summary of the regression and performance values extracted from 1-2-gram and 1-3-gram with variable number of neurons in the hidden layer. Table 8 gives a summary of the regression and performance values extracted from 1-2-gram and 2-3-gram with variable number of neurons in the hidden layer. Table 9 gives a summary of the regression and performance values extracted from 1 2-gram and 1-2-3-gram with variable number of neurons in the hidden layer. Table 1, Table 2 and Table 3 show the percentages (ratios) from Affymetrix 1 dataset of nucleotides, di nucleotides and tri nucleotides respectively. Using pattern recognition toolkit to investigate the behavior of our predictions in terms of confusion matrices (CM) and receiver operating characteristic (ROC) curves, the results with 1-3-gram are shown in Table 10. The results with 2-3-gram and 1-2-3-gram (not shown) are not as good as those obtained using 1-3-gram.

## Discussion

As noted in 15, the absolute set comprises 4 x 594 values, where the four values are the absolute signal strengths of the bases [ACGT] on each of 594 lines. Absolute signal strengths are normalized to values between 0.0 - 1.0, from which the Neural Network/n-gram process predicts values (= 0.0 - 1.0). The Prediction set correctly identifies the highest value (1.0) in the normalized set for all 594 lines, which is, of course, the highest value and therefore the correct base call in the absolute set. This is not necessarily a trivial result, as the predictive function must accommodate all targets in the 4 x 594 sets. Using regression toolkit, we observed that the values in Table 4, Table 5 and Table 6 were generally better than the results obtained in 15 where 1-2-gram composition of the n-grams were used. This is in part due to the increment in the n-grams from 2 (two) to 3 (three). A look at Table 7, Table 8 and Table 9 shows reduction in the validation error and increment in the regression value when we compare the respective n-gram compositions. The average best validation performance (Bvp) and regression values obtained in 15 for 1-2- gram was 0.002793 which translated to 99.72% accuracy with average regression value of 0.98803. These numbers decreased (increased) to 0.002499 and 0.99112 when we used the 1-3-gram com- position. Again, a comparison of 1-2-gram and 2-3-gram showed a decrease in best validation performance to 0.002253 and increase in the regression value to 0.99180 for the 2-3-gram. In the case of 1-2-3 gram, the best validation performance value again decreased to 0.002056 or 26.4% when compared with the value obtained with 1- 2-gram. The regression value also increased to 0.99191 from 0.98803 obtained with 1-2-gram. This is again due to the increment in the n-gram number. The use of pattern recognition toolkit to investigate the behaviour of the confusion matrices and receiver operating characteristic (ROC) curves showed general confusion matrices value of 99.8% using 40 neurons in the hidden as shown in Table 10 and the points in ROC curve lying in the upper left corner. These are good signs of near expected results. 

## Conclusion

 We can predict the signal intensities via their normalized values from Affymetrix data using artificial neural network based n-gram model. It seems the higher the n-gram value and appropriate composition, the better the predictive accuracy of the model. The usage of higher n-gram values and their different compositions are considered in this paper. Efforts could be made to increase the number of n-grams to see if better results can be obtained which we envisage to be true. An effort could also be made to get optimal number of neurons in the hidden layer that give maximal regression values and lower mean square error. An increase in regression value to say 0.999 is indicative of a much better prediction with its attendant low mean square errors which is a measure of performance. As we increase the n-grams, we can also check which composition of the n-grams give better results. Greater confusion matrix values along the diagonal and ROC curves points in the upper most left corner can also be achieved for better classification.

## Figures and Tables

**Table 1:** The four nucleotides and their percentages (ratios)

Nucleotides	Percentage
A	0.31
C	0.31
G	0.13
T	0.25

**Table 2:** The sixteen di nucleotides and their percentages (ratios)

Di nucleotides	Percentage	Di nucleotide	Percentage
AA	0.1	GA	0.04
AC	0.09	GC	0.04
AG	0.05	GG	0.03
AT	0.07	GT	0.03
CA	0.09	TA	0.08
CC	0.11	TC	0
CG	0.03	TG	0.03
CT	0.09	TT	0.06

**Table 3:** The 64 tri nucleotides and their percentages (ratios)

Tri nucleotides	Percentage	Tri nucleotides	Percentage
AAA	0.032	CAA	0.028
AAC	0.03	CAC	0.027
AAG	0.013	CAG	0.012
AAT	0.023	CAT	0.025
ACA	0.026	CCA	0.027
ACC	0.031	CCC	0.036
ACG	0.007	CCG	0.008
ACT	0.025	CCT	0.034
AGA	0.011	CGA	0.008
AGC	0.017	CGC	0.009
AGG	0.011	CGG	0.005
AGT	0.01	CGT	0.005
ATA	0.022	CTA	0.032
ATC	0.022	CTC	0.023
ATG	0.01	CTG	0.011
ATT	0.02	CTT	0.02
GAA	0.012	TAA	0.025
GAC	0.01	TAC	0.023
GAG	0.008	TAG	0.016
GAT	0.007	TAT	0.02
GCA	0.012	TCA	0.025
GCC	0.017	TCC	0.022
GCG	0.003	TCG	0.007
GCT	0.011	TCT	0.019
GGA	0.007	TGA	0.012
GGC	0.01	TGC	0.007
GGG	0.004	TGG	0.006
GGT	0.005	TGT	0.006
GTA	0.009	TTA	0.02
GTC	0.006	TTC	0.019
GTG	0.003	TTG	0.007
GTT	0.006	TTT	0.015

**Table 4:** Best validation performance (Bvp) and regression values with 1-3-gram with varying number of neurons in the hidden layer

No. of neurons	Regression values	Best validation performance
20	0.99081	0.002713
25	0.99135	0.002539
30	0.99115	0.00247
40	0.99117	0.002275
Averages	0.99112	0.002499

**Table 5:** Best validation performance (Bvp) and regression values with 2-3-gram with varying number of neurons in the hidden layer

No. of neurons	Regression values	Best validation performance
20	0.99171	0.002199
25	0.99181	0.002192
30	0.99183	0.002263
40	0.99207	0.00236
Averages	0.9918	0.002253

**Table 6:** Best performance and regression values with 1-2-3-gram with varying number of neurons in the hidden layer

No. of neurons	Regression values	Best validation performance
20	0.99171	0.002199
25	0.99181	0.002192
30	0.99183	0.002263
40	0.99207	0.00236
Averages	0.9918	0.002253

**Table 7:** Regression and best validation performance (Bvp) values for 1-2-gram and 1-3-gram with varying number of neurons in the hidden layer

No. of neurons	R-values		Bvp values	
	1-2-gram	1-3-gram	1-2-gram	1-3-gram
20	0.9884	0.99081	0.002849	0.002713
25	0.98914	0.99135	0.002666	0.002539
30	0.98874	0.99115	0.00313	0.00247
40	0.98587	0.99117	0.002525	0.002275
Average	0.98803	0.99112	0.002793	0.002499

**Table 8:** Regression and best validation performance (Bvp) values for 1-2-gram and 2-3-gram with varying number of neurons in the hidden layer

No. of neurons	R-values		Bvp values	
	1-2-gram	2-3-gram	1-2-gram	2-3-gram
20	0.9884	0.99171	0.002849	0.002199
25	0.98914	0.99181	0.002666	0.002192
30	0.98874	0.99183	0.00313	0.002263
40	0.98587	0.99207	0.002525	0.00236

**Table 9:** Regression and best validation performance values for 1-2- gram and 1-2-3-gram with varying number of neurons in the hidden layer

No. of neurons	R-values		Bvp values	
	1-2-gram	1-2-3-gram	1-2-gram	1-2-3-gram
20	0.9884	0.99185	0.002849	0.00208
25	0.98914	0.99187	0.002666	0.00203
30	0.98874	0.99191	0.00313	0.00213
40	0.98587	0.992	0.002525	0.00213
Average	0.98803	0.99191	0.002793	0.002056

**Table 10:** Confusion matrix values and ROC curves dynamics for 1-3 gram

No. of neurons	Overall CM values %	Bvp	ROC curve dynamics
20	91.2	0.04239	Upper left corner
25	97.1	0.025996	Upper left corner
30	99.8	0.007321	Upper left corner
40	99.8	0.003937	Upper left corner
